# Letter from New York City

**Published:** 1902-04

**Authors:** 


### Domestic Correspondence.

### LETTER FROM NEW YORK CITY.

TO THE EDITOR:

SIR,—The many members of the New York College of Dentistry, and also adjacent cities, who were invited to attend the lecture and reception on February 4, given by the New York College of Dentistry at the college to Professor T. E. Weeks, of Minneapolis, certainly felt fully repaid for attending. The subject was "Cavity Preparation," which was presented in an interesting and scientific manner by aid of large tooth models, enlarged microscopic lantern slides, and excavators. The vital importance of properly preparing enamel margins and of so shaping the cavity for reception and retention of filling that the great stress or strain materially imposed on a filling, in its usage, would be sustained rather by the flat floor or large part of the cavity than by the walls, the occlusal surface also being utilized in instances of approximal fillings, was demonstrated carefully and at length. To Professor G. V. Black was accorded the honor of having successfully systematized the respective portions of the tooth-structure into a simple dental nomenclature. The subject of "extension for prevention" Professor Weeks discussed, his views being conservative on the subject. He came to the conclusion that the operator, in each individual case presented, should with careful judgment decide as to what degree or extent the enamel should be trimmed and cavity enlarged to retain the filling. After the lantern-slides were shown, demonstrating clearly enamel-rods under different forms of enamel chiselling in preparation for filling, a collation was served.

The subject at the meeting of the First District Dental Society of New York was "Practical Hints and Suggestions," by Dr. Chas. E. Francis, of New York.

A large number were present on this Tuesday evening, February 11, to hear Dr. Francis read a very interesting paper on the above subject. The paper was especially valuable to the younger men present, as well as to the older dentists, the remarks coming from one who I understand originally introduced this important subject many years ago, and who now after a long and honorable career in the practice of dentistry, extending over a period of half a century, was at last prevailed upon to come forth from his retirement and impart to those present his observations upon this all-important subject. He dwelt upon the importance of the personal appearance of the dentist, not only as regards his attire, but also his address, his desire to please and be interested in every individual patient, be they rich or otherwise, at the same time maintaining a professional dignity towards each patient. His remarks were interspersed with interesting personal experiences and reminiscences, which were very uncommon, for the doctor on first coming to our city to practise came by boat, there being no railroad connections with New York at that time, and only three lines of stages in the city. The charm and interest in an address of this character was greatly enhanced by his own delivery of it. Dr. Francis must, indeed, have felt highly gratified after hearing so many warm expressions of appreciation at the close of his address by the older practitioners who were present.

The afternoon clinics were full of interest, Dr. G. Lenox Curtis performing an alveolar operation by use of cocaine, which, by the way, Dr. Curtis says is made practically safe if volasen, ten or fifteen drops in water, is given to the patient prior to the operation.

Dr. Wm. Carr urged those present to communicate with their respective assemblymen at Albany, for the purpose of defeating a certain measure introduced there, which would not have for its object the best interests of the profession at heart.

It was announced that Dr. T. W. Brophy, of Chicago, would read a paper on "Surgical Treatment of Congenital Defects of the Palate." All were invited, on February 26, at "The Academy."

The regular meeting of The New York Institute of Stomatology was postponed until Wednesday, February 12, Lincoln's birthday. Dr. F. Park Lewis, M.D., of Buffalo, read a paper on "Adenoids," the meeting being at Dr. E. A. Bogue's office. Several physicians were invited to discuss the paper, which was interesting. Dr. Lewis explained that adenoids were an hypertrophied condition of lymphoid tissues normally found in the vault of the pharynx posterior to the nasal opening. He defined their structure, and discussed their relation to the general system. The subject was presented in a clear and practicable manner. Among other things of importance, he said that a relationship between face, nose, mouth, and chest deformities was most fully recognized by the medical profession where adenoids were present, improper or difficult breathing causing anæmic blood conditions due to lack of proper oxygenation, etc., eventually giving rise to many disturbances or diseases, in addition to the deformities previously noted; that the element of heredity enters largely into adenoid growths, and that adenoids atrophy somewhat at puberty. He cited instances where many operations have been performed on new-born infants. He also dwelt upon the inflammatory catarrhal conditions arising from adenoid obstruction, and of many other conditions bearing on the subject. The discussion which followed evidenced a very careful consideration of this subject by every progressive practitioner.

Incidents of office practice followed the paper of the evening, and the subject of the different varieties of wood was discussed for use in cleaning and polishing teeth, orange, cedar, and hard maple being among those advocated. A simple porte-polisher was presented by Dr. F. M. Smith, through which a hole was made at the end of the instrument, into which a stick could be forced, and by having several instruments any angle desired could be had. A tooth-brush consisting of alternately longitudinal layers of bristles and badger-hairs, the latter being longer, for the purpose of brushing inflamed gums, was also presented.

A collation followed the evening session.

The twenty-second annual meeting of the Central Dental Association of Northern New Jersey was held in Newark, on February 15. After the banquet, at which one hundred and fifty were present, speech-making followed. The president gave an address of welcome to those present. Among the important remarks he made was one referring to a resolution adopted to found a home where original research, examination of the saliva, bacteriology, etc., was to be pursued by aid of proper scientific instruments.

On Tuesday, February 18, the regular meeting of the New York Odontological Society was held. Dr. D. D. Smith, of Philadelphia, gave a very interesting paper on tightening loose lower incisors and cuspids by means of thorough and systematic treatment. Among the points Dr. Smith touched upon, after, of course, the thorough removal of all deposits on the teeth, was that for the devitalization of the necessary teeth in work of this character. He used arsenic, adding that he had no hesitancy in removing pulps. The arsenic when applied and allowed to remain for forty-eight hours in tissue over a pulp, the latter not exposed, would thoroughly devitalize the pulp, after the removal of which the root was treated with wood creosote and allowed to remain for twenty-four hours. The changes occurring after pulp-extraction were discussed, Dr. Smith maintaining that the root was strengthened by the operation. The method advocated by him of holding or strengthening weaker teeth was by removing pulps in adjacent teeth, fitting posts in the canal to which were attached a soldered cap to the palatal side of the teeth, no gold showing whatever, and joined or supported by soldering. His method could not be described in a few words. Dr. S. G. Perry and others followed in the discussion, he among others expressing the excellent results he had attained by using creosote, and filling with oxychloride, as did Dr. Smith. Dr. Rhein advocated sodium, potassium, and other medicines, considering them more scientific according to researches than creosote, which was characterized as an embalming rather than a germicidal property. Dr. D. D. Smith closed the discussion. A vote of thanks was tendered him for his able paper, and the meeting adjourned.

Dr. Brophy's address on "Surgical Treatment of Congenital Defects of the Palate" was delivered before the New York Academy of Medicine on February 26. It was ably presented by aid of stereopticon slides. A large number of dentists were invited to be present. He cited among other things that, of six hundred cases operated upon, two hundred and fifty had been successful. The importance of performing the operation at an early age was dwelt upon, and that before the child is six months old, carefully describing the changes occurring in such an operation. Many prominent men joined in the discussion that followed.

LOCHINVAR.

